# ASO Author Reflections: Bridging the Gap in Palliative Therapy for Young Adults with Advanced Gastrointestinal Cancer

**DOI:** 10.1245/s10434-025-17190-3

**Published:** 2025-03-26

**Authors:** Olivia Monton, Kimberly Kopecky, Fabian M. Johnston

**Affiliations:** 1 Department of Surgery, Johns Hopkins University School of Medicine, Baltimore, MD USA; 2 Department of Epidemiology, Johns Hopkins Bloomberg School of Public Health, Baltimore, MD USA; 3 Department of Surgery, McMaster University, Hamilton, ON Canada; 4 Division of Surgical Oncology, Department of Surgery, University of Alabama at Birmingham Heersink School of Medicine, Birmingham, AL USA; 5 Department of Surgery, Wake Forest University School of Medicine, Winston-Salem, NC USA

## Past

Advanced gastrointestinal (GI) cancer imposes a significant symptom burden, with young adults (YAs) facing unique challenges, including heightened psychological distress.^1^ Palliative therapy plays an important role in managing symptoms, improving quality of life, and addressing psychosocial needs.^2^ However, little is known about its use among YAs with advanced GI cancer.

## Present

This retrospective cohort study analyzed data from the National Cancer Database (NCDB), including data for 43,616 YAs with advanced GI cancer diagnosed between 2004 and 2020.^3^ The majority of the patients (*n* = 39,796, 91.24%) did not receive palliative therapy, underscoring a critical gap in cancer care. Although the use of palliative therapy has increased over time (from 5.33% in 2004 to 12.36% in 2020; *p*_trend_ < 0.05), overall rates remain low, particularly compared to those of older adults (8.76% vs 12.83%). This trend is concerning because YAs, who often have complex physical and psychosocial needs, stand to benefit significantly from palliative therapy.^4^ The low uptake may reflect a preference for aggressive, curative-intent treatment in this younger, and generally healthier patient population.^5^

## Future

Addressing this gap requires several key steps. First, expanding the evidence base on palliative therapy use among YAs across different cancer types is essential. Second, more comprehensive data on palliative care delivery and associated outcomes are needed to better understand utilization patterns, trends, and patient-centered outcomes in more detail. Finally, qualitative research is necessary to explore the barriers to and benefits of palliative-intent treatment for YAs with advanced GI cancer. These efforts may inform the development of targeted interventions aimed at improving the uptake and use of palliative therapy among this vulnerable and underserviced population.

## Disclosure

There are no conflicts of interest.
